# Automated Segmentation and Connectivity Analysis for Normal Pressure Hydrocephalus

**DOI:** 10.34133/2022/9783128

**Published:** 2022-01-09

**Authors:** Angela Zhang, Amil Khan, Saisidharth Majeti, Judy Pham, Christopher Nguyen, Peter Tran, Vikram Iyer, Ashutosh Shelat, Jefferson Chen, B. S. Manjunath

**Affiliations:** ^1^Vision Research Laboratory, Electrical and Computer Engineering, University of California, Santa Barbara, Santa Barbara, CA, USA; ^2^Chen Lab, Department of Neurosurgery, University of California, Irvine Medical Center, Orange, CA, USA; ^3^Santa Barbara Cottage Hospital, Santa Barbara, CA, USA

## Abstract

*Objective and Impact Statement*. We propose an automated method of predicting Normal Pressure Hydrocephalus (NPH) from CT scans. A deep convolutional network segments regions of interest from the scans. These regions are then combined with MRI information to predict NPH. To our knowledge, this is the first method which automatically predicts NPH from CT scans and incorporates diffusion tractography information for prediction. *Introduction*. Due to their low cost and high versatility, CT scans are often used in NPH diagnosis. No well-defined and effective protocol currently exists for analysis of CT scans for NPH. Evans’ index, an approximation of the ventricle to brain volume using one 2D image slice, has been proposed but is not robust. The proposed approach is an effective way to quantify regions of interest and offers a computational method for predicting NPH. *Methods*. We propose a novel method to predict NPH by combining regions of interest segmented from CT scans with connectome data to compute features which capture the impact of enlarged ventricles by excluding fiber tracts passing through these regions. The segmentation and network features are used to train a model for NPH prediction. *Results*. Our method outperforms the current state-of-the-art by 9 precision points and 29 recall points. Our segmentation model outperforms the current state-of-the-art in segmenting the ventricle, gray-white matter, and subarachnoid space in CT scans. *Conclusion*. Our experimental results demonstrate that fast and accurate volumetric segmentation of CT brain scans can help improve the NPH diagnosis process, and network properties can increase NPH prediction accuracy.

## 1. Introduction

**Figure 1:**
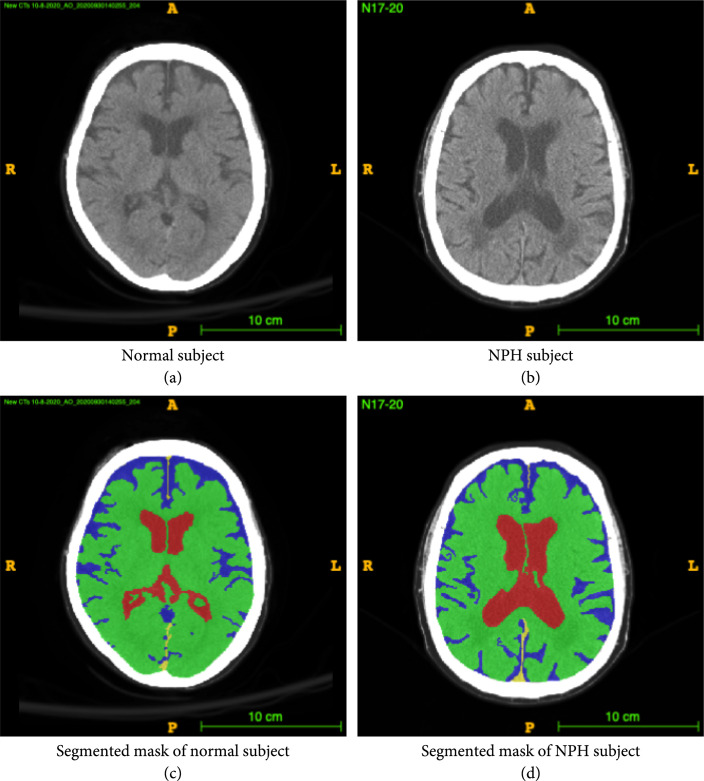
Example CT image slice of area with widest frontal horns in (a) a normal subject and (b) a subject diagnosed with NPH. (c) and (d) are masks overlaid on the original image indicating the ventricles (red), gray-white matter (green), and subarachnoid space (blue). In this example, the width of the frontal horns appear similar in the normal subject and the NPH subject, but the total volume of the ventricles differs. This is one example of a case where 3D volumetric measurements may be helpful in differentiating potential patients with NPH from those without NPH. These annotations are carefully created from the 3D data and are available to the public.

This paper describes a novel and robust method for segmenting Computed Tomography (CT) scans of the brain and predicting possible Normal Pressure Hydrocephalus (NPH) from the segmentation. More than an estimated 700,000 Americans have NPH, but due to the presence of confounding comorbidities and the lack of a rigorous diagnosis protocol, the majority of cases are under- or misdiagnosed [1] as other forms of often comorbid dementia, leading to a delay in treatment that would significantly improve neurologic function. NPH is one of few reversible causes of dementia in the elderly, making correct diagnosis important, as shunt placement to drain the excess cerebrospinal fluid (CSF) has been demonstrated to be a safe and effective treatment [2]. Meanwhile, misdiagnosis and missed treatment can lead to decreased quality of life and cognitive deterioration. NPH presents as enlargement of the lateral ventricles in the brain, while maintaining normal CSF pressure levels. CSF normally flows through the subarachnoid space and ventricles, but in patients with NPH, there is an abnormality of CSF flow and absorption that results in CSF accumulation in the ventricular system, causing the ventricles to swell, as illustrated in Figure 1. This swelling causes a multitude of neurological symptoms if left untreated.

NPH afflicts mainly elderly patients, often with comorbid factors such as Parkinson’s or Alzheimer’s disease, and is commonly accompanied by symptoms of dementia. More specifically, cognitive dysfunction, changes in gait, and urinary incontinence are major symptoms indicating the presence of NPH [2]. Current diagnostic methods for NPH involve a mixture of clinical and imaging approaches [2]. Most commonly, a memory test is conducted, along with observation of gait and inquiry of urinary continence status. In addition, a Computed Tomography (CT) scan is often acquired to visually determine lateral ventricle size.

**Figure 2:**
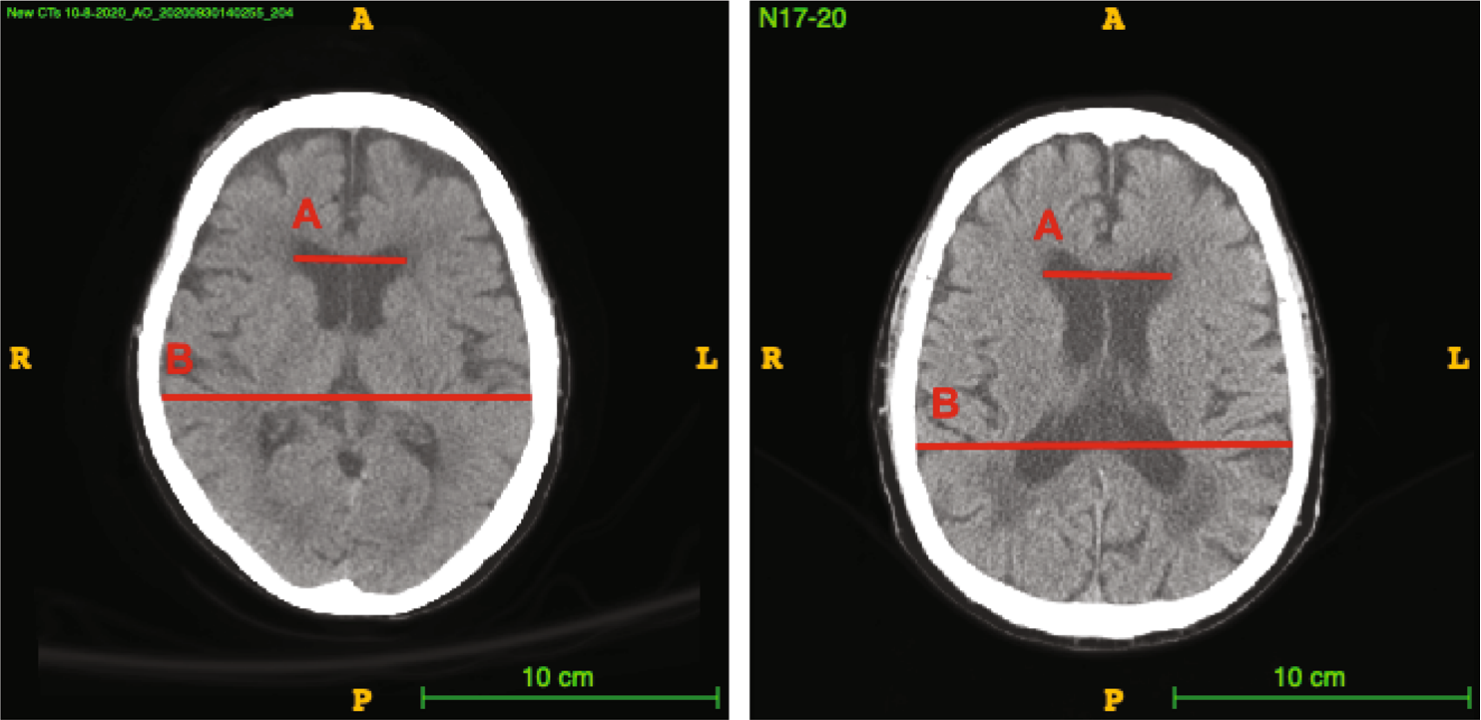
Demonstration of the measurements involved in obtaining Evans’ index in a normal (a) and an NPH (b) patient. A is the distance between the widest part of the frontal horns. B is the width of the widest part of the skull. Evans’ index is computed as A/B.

Sometimes, Evans’ index, a 2D manually computed ratio illustrated in Figure 2, is computed as a proxy for the size of the lateral ventricles in comparison to the size of the brain as a whole. Evan’s index is the ratio of the transverse diameter of the anterior horns of the lateral ventricles to the greatest internal diameter of the skull in a single slice of a 3D volume CT or Magnetic Resonance Imaging (MRI) scan.

Current guidelines define ventricular enlargement with an Evan’s index of greater than 0.3 [3]. However, determining Evans’ index is time-intensive, manual, and prone to error due to varying imaging conditions and subjectivity of measurement. It has been shown that Evan’s ratio in fact varies greatly depending on the level (slice location) of the brain CT scan image at which the frontal horns and maximal inner skull diameters are measured [3]. More importantly, the Evans’ index measurement only takes into account a proxy for the ventricle and skull sizes, as shown in Figure 2, and does not account for other important volumes such as the subarachnoid space—the smaller folded channels also containing CSF located near the surface of the brain—and gray-white matter volumes. These volumes are important due to the presence of increased subarachnoid space and decreased gray-white matter in subjects with dementia, which is a major confounding factor in diagnosing NPH.

A 3-dimensional multiclass volumetric method of measuring the relevant regions of the brain could help to mitigate these challenges and holds promise for improving NPH differential diagnosis [4]. Manual 3D segmentation of CT scans is a time-consuming process, with one detailed volumetric segmentation of the ventricles, subarachnoid space, and gray-white matter taking up to twenty hours to complete. Moreover, delineating these regions in detail requires domain expertise, limiting the possibility of such segmentation being done on a regular basis. An automated method to segment CT scans would allow for widespread usage of volumetric analysis as an aid in NPH diagnosis, as well as the monitoring of patients over time to determine the effects of treatment.

An automated method of computing Evan’s ratio from CT is presented in [5], but this method loses the volumetric advantage of directly computing the volumes from CT scans. For segmentation of the lateral ventricles, Coupé et al. [6] use expert priors to aid in patch-based segmentation of the lateral ventricles in MRI. Yepes-Calderon et al. [7] implements a method for automated ventricular volume measurement in MRI using the strong force algorithm to find the best statistical features and a support vector machine to compute the final classification. The paper claims feasibility in CT but does not discuss NPH. Another method of lateral ventricle segmentation in MRI is presented in [8] using a fuzzy representative line. The authors in [9] explore challenges in ventricle segmentation from MRI using neural networks, finding difficulties transferring segmentation models trained normal patients to work with NPH patients. Cai et al. [10] perform highly detailed segmentation of CT scans for the purpose of NPH analysis, using 4-6 slices per scan to capture the ventricular region of the scan.

To our knowledge, our proposed method is the first to predict NPH by first fully segmenting the subarachnoid space, lateral ventricles, and gray-white matter of the brain from a CT scan. An automated method of segmenting all of the relevant regions in the brain from a CT scan would greatly aid in the discovery and treatment of patients with NPH. Moreover, changes in brain connectivity due to the enlarged lateral ventricles may be modeled from this segmentation and its effects used to aid in NPH prediction.

The connectome is a map of the white tissue structures connecting different regions in the brain, which consist of axonal bundles that neurons use to communicate with one another. These white tissue structures can be seen using diffusion MRI, an imaging method which allows us to see the diffusion properties of water through the matter being imaged. Since water travels in a directed pattern through axonal bundles, they are visible through this method. Diffusion orientation computation methods and fiber tracking (Yeh and Tseng [11]; Yeh et al. [12]) reveal network properties of the brain that are unique to each connectome, in a process called diffusion tractography.

We hypothesize that the enlargement of the lateral ventricles in patients with NPH cause changes to the connectome, especially in the regions closest to the enlarged ventricular space (i.e., the frontal horns). Since patient-specific diffusion imaging is not gathered in a typical NPH clinical workflow, we propose an inventive method to approximate changes in the connectome using the segmented scan and a diffusion MRI atlas. The network metric derived from this approximated connectome is used as additional information during the NPH prediction process. To our knowledge, this is the first time connectome information has been combined with volumetric segmentation features to predict the presence of a disorder such as NPH.

The major contributions of this paper are as follows.

(1) We have developed a robust, data-driven CT segmentation method which is derived from data from NPH and age-matched normal patients which can aid in NPH diagnosis. Our algorithm is the first of its kind to take into account the spatial distribution of the regions of interest. The robustness of our algorithm allows for the segmentation of CT scans from both normal and NPH subjects, which has historically been a challenge even with deep learning (Shao et al. [9])
(2) Additionally, we combine diffusion tractography information with the segmentation in a novel way to improve NPH prediction
(3) The creation of a novel and valuable dataset of detailed and full manual segmentations of CT brain scans. This data will be made publicly available and can further benefit the medical research community. The code is available on GitHub at https://github.com/UCSB-VRL/NPH_Prediction/tree/connectome

## 2. Data

### 2.1. Data Collection

To study the morphological differences between NPH and non-NPH patients and create a pipeline for predicting NPH, CT brain scans are collected from patients with and without NPH. To incorporate additional white matter structural (diffusion tractography) information into the NPH prediction process, diffusion MRI of normal subjects between the ages of 75 and 85 are collected from the Alzheimer’s Disease Neuroimaging Initiative (ADNI).

The CT data comes from two sources: the University of California Irvine Medical Center (UCI) and the Santa Barbara Cottage Hospital. This is a retrospective study, with all images deidentified as specified by the IRB agreement between each medical center and the University of California, Santa Barbara.

There is no protocol determining the number of slices, orientation, or other imaging parameters for the data used in this study. CT scans of n=65 subjects are included in the study, with 42 subjects having a diagnosis of normal and 23 subjects having a diagnosis of NPH. Axial scans are acquired as part of the treatment process, and the number of slices varied from 25 to 207. For the subjects from UCI, the average subject age is 75±15 years. For the subjects from Cottage Hospital, the average age of the subjects is 72±14 years.

### 2.2. Data Processing and Annotation

40 manual segmentations are performed by members of the research team under direct supervision and validation by a neurological surgeon. Each annotation took approximately 20 hours to complete and includes the subarachnoid space, ventricles, gray-white matter, and cerebellum. The annotation is first initialized using semisupervised snake segmentation through the ITK-SNAP tool (Yushkevich et al. [13]), then further refined manually. There are a total of approximately 2,000 completed manually segmented slices used in this study, taking approximately 800 hours to annotate. The rest of the scans are not used for segmentation training or validation but are added to the dataset for diagnosis training and validation.

For annotation consistency using ITK-SNAP, the viewing window is adjusted to have a viewing intensity minimum of −980 and maximum of 80. After window adjustment, curve-based contrast adjustment is done with 3 control points. The middle control point is set at x=−10 and y=0.020.

The refined annotation is compared to the initial semisupervised segmentation. Evan’s index, measured under direct supervision of a neurological surgeon, is calculated for all subjects.

As noted earlier, diffusion tractography information is incorporated in a novel way into the NPH prediction process to improve the prediction results. In addition to the CT data, diffusion MRI of 11 normal subjects between the ages of 75 and 85 from the Alzheimer’s Disease Neuroimaging Initiative (ADNI) are used to create an average connectome with which to conduct connectivity studies comparing NPH and non-NPH subjects.

## 3. Approach

### 3.1. Algorithm Overview

**Figure 3:**
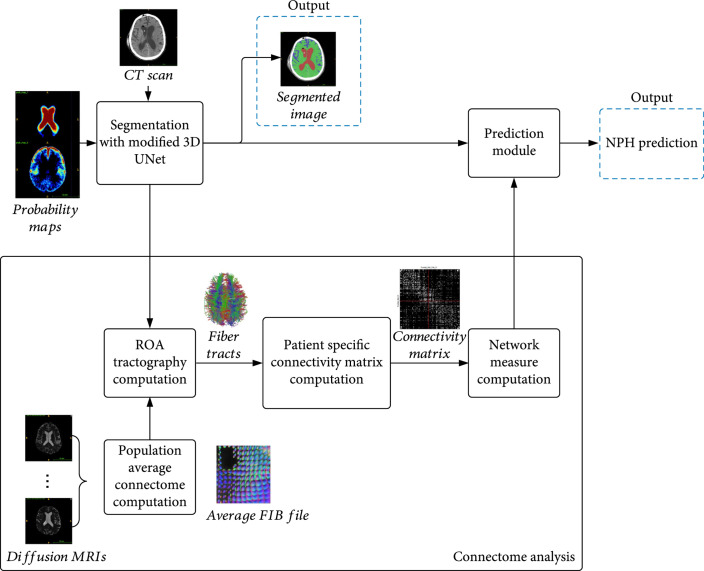
Flowchart for CT segmentation and NPH prediction. Segmentation of the CT scan is achieved with a modified 3D UNet. The segmentation result is used to create a Region of Avoidance (ROA) during the computation of the average connectome of normal elderly people. From the computed tractography results, a patient-specific connectivity map is created, and the associated network properties are used in combination with the features from the modified 3D UNet (prediction module) to reach a final prediction of probable NPH.

**Figure 4:**
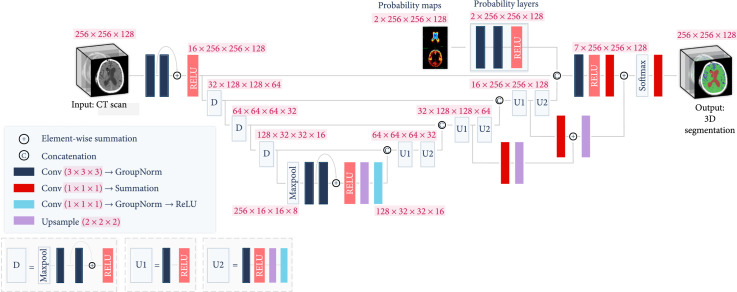
Modified 3D UNet. The 3D CT scan is fed into a UNet, with the standard downsampling and upsampling layers. The layer with aggregated probability maps improves the performance of the network for the ventricle and especially for the subarachnoid class.

First, the CT image is segmented into three regions of interest (ROI)—the gray-white matter, subarachnoid space, and lateral ventricles—using a modified trained 3D UNet. These ROIs are important to NPH prediction due to the enlargement of the lateral ventricles in relation to the rest of the brain which occurs during NPH. The UNet uses both the original CT scan and a probability map created from the ground truth annotations to provide contextual data to the upsampling layers. The segmented volumes are used to create a subject-specific connectome from average older subject diffusion MRI tractography. The segmented volumetric and diffusion tractography information are used as input features to a fully connected layer to perform feature fusion. The fused features are then used to make the final prediction of NPH vs. non-NPH. Figure 3 illustrates the main components of the prediction process. Figure 4 shows the layers in the modified 3D UNet. Algorithm 1 summarizes the various steps.

**Algorithm 1: NPH prediction. CT scan segmentation.**
 **Input:** CT scan of the subject, MNI152 Atlas, and population average diffusion data for diffusion tractography computation in the MNI152 space
 **Output:** 3D segmentation of CT scan into 3 regions, and NPH score
1 Compute the affine transform matrix H that aligns the MNI152 atlas to the subject space
2 Compute probability maps using ground truth annotations and transform it into the subject space using H
3 The CT scans are segmented using modified 3D UNet and affine transformed probability maps. The resulting segmentation is mapped back to the MNI152 space using $H^{-1}$ for the tractography computations below
4 Compute ROA (Region of Avoidance) tractographs using the ventricles as the ROA
5 Compute patient-specific connectivity matrix and the derived network properties
6 Using the segmentation volumes and network properties, predict whether the subject has NPH

### 3.2. Segmentation Using a Modified 3D UNet

We modified a 3D UNet architecture to perform accurate segmentations of the lateral ventricles, subarachnoid space, and gray-white matter in Computed Tomography (CT) scans of the brain. The relative values in each CT scan are preserved to maintain density information. In preparation for the next steps, an affine transform matrix is computed by matching the features of the CT scan to an MRI MNI152 atlas using FSL FLIRT (Jenkinson et al. [14]). The original image is then passed to a modified 3D UNet, along with a probability map derived from the transformed volume which is further described in Section 3.2.

Three-dimensional convolutional neural network models are currently the state-of-the-art for medical image segmentation. Because many of the most successful models are variants of 3D UNet (Bui et al. [15]; Kao et al. [16]), we have adopted it as the basic model from which we create a segmentation pipeline. The full model is shown in Figure 4. The input size is 256×256×128. There are 4 downsampling blocks each containing two 3×3×3 convolutional layers and one rectified linear unit (RELU) (Agarap [17]). These blocks each reduce the dimensions of the previous input by half while doubling the number of feature layers. The last downsampling block is preceded by max pooling. Following the downsampling layers, there are 4 upsampling layers. Each upsampling layer consists of 2 iterations of a 3×3×3 convolutional layer followed by a RELU, a 2×2×2 upsampling layer, a 1×1×1 convolutional layer, Group Normalization (Wu and He [18]), and another RELU. Each upsampling block increases the dimensions by 2 in all 3 dimensions, while halving the number of feature layers. The output of each upsampling block is concatenated with the output of the corresponding downsampling block. For the entire network, each 3×3×3 convolutional layer is followed by group normalization. After the first RELU of the 2nd and 3rd upsampling blocks, the output at that layer is passed through a 1×1×1 convolutional layer, followed by summation and a 2×2×2 upsampling layer. The output of these extra blocks are concatenated with the next layer as shown in Figure 4. The output of the final upsampling block is also concatenated with the output of the probability map layers (described in the next paragraph) and passed through one more 3×3×3 convolutional layer, a RELU, and one 1×1×1 layer. Finally, the end result is passed through a softmax layer and a 1×1×1 convolution layer followed by summation. The final output is the 3D segmentation of the input CT scan, at a size of 256×256×128. Our main modification to the 3D UNet is the probability map layers, which are described in the next paragraph.

#### 3.2.1. Probability Maps

While the base 3D UNet model provided good results for brain CT segmentation, the trained network still failed to differentiate between subarachnoid space and ventricle on some border regions. In addition to this, subarachnoid space would often be misclassified as gray-white matter if the subarachnoid spaces are thin, as the intensity values are lighter for thin subarachnoid spaces. Since the occurrence of subarachnoid space and ventricles are highly similar between subjects, a location-aware layer is added to the network to boost performance in these areas. Regional probability maps are generated from the ground truth annotations and used in the upsampling layer of the 3D UNet. The probability maps are created by averaging all of the ground truth annotations once they have been registered to a common space (MNI152). The same probability map is used for each patient, but it is transformed into that patient’s space using a reverse affine transformation that is calculated through the registration process. Two convolutional layers and a RELU are used to learn the weights with which to apply each probability map, and the output of the RELU is concatenated to the output features of the 3D UNet at the last upsampling layer. The dimensions of the input and output to the probability maps layers are 2×256×256×128, with the 2 being the number of probability maps we are using (corresponding to the ventricle and subarachnoid space). To see the probability map layers, see Figure 4. These probability maps are then transformed into the patient space with an affine transformation. We use the ground truth annotations to create spatial probability maps for each of the 2 significant regions—ventricles and subarachnoid spaces. Thus, each voxel location in the probability map represents the likelihood of that voxel belonging to one of these regions.

### 3.3. Diffusion Tractography Analysis

#### 3.3.1. Population Average Connectome Computation

Since the swelling of the ventricles that occur during NPH may result in damaged white matter tracts that affect brain connectivity as a whole, we analyzed the effect of using white matter disturbance information in the NPH prediction process. However, diffusion MRI are not commonly collected from patients with suspected NPH in a clinical setting. Therefore, we used a proxy for the possible white matter disturbances by modifying the average diffusion tractography from normal (control) older subjects in the ADNI dataset with the automated segmentation outputs of each subject. Deterministic fiber tractography using QSDR reconstruction (Yeh and Tseng [11]) is conducted of the average diffusion tractography from 11 subjects in the ADNI dataset with a seed count of one million seeds per sample. This results in approximately 50,000 tracts per subject.

#### 3.3.2. ROA Tractography Computation

For each subject in the NPH dataset, the predicted ventricle segmentation is used as a Region of Avoidance (ROA) during fiber tractography. Affine transformations are computed using FSL (Jenkinson and Smith [19]; Jenkinson et al. [20]) by first thresholding the skull and computing a rigid transformation from each CT scan to an MNI152 MRI atlas, then an affine transform using the soft tissue inside of the brain. The affine matrix for transforming each CT to MNI152 space is then used on the ventricle segmentation for each subject scan to project the ROA into MNI152 space for comparison.

#### 3.3.3. Patient-Specific Connectivity Matrix and Network Metric Computation

A 90×90 connectivity matrix computed using the Automated Anatomical Labeling 2 (AAL2) atlas (Tzourio-Mazoyer et al. [21]) in MNI152 space is constructed for each set of fibers, and the network properties of the matrix are used as additional features to a shallow, fully connected network which also uses the features from the last downsampling layer of the UNet to differentiate NPH and non-NPH subjects. These network properties are briefly described in Table 1 and are explained in detail by Bullmore and Sporns [22].

**Table 1:** Network coefficients used for NPH vs. non-NPH comparison. All coefficients included a weighted and a binary version, except for the network density.

Network coefficients	Brief description
Density	Fraction of present connections to possible connections
Clustering coeff. average	Fraction of triangles (node’s neighbors that are also neighbors of each other) around a node (vertex connected to other vertices)
Transitivity	Ratio of triangles to triplets (three nodes that are connected by either two or three ties) in the network
Network characteristic path length	Average shortest path length in the network
Small-worldness	Average path length of the network divided by the average path length of a random network with the same node and edge (connection between two nodes) count as the network being analyzed
Global efficiency	Average efficiency (1/distance) between all sets of nodes
Diameter of graph	Maximum eccentricity (maximal shortest path length between a node and any other node)
Radius of graph	Minimum eccentricity
Assortativity coefficient	Correlation of the degree (number of overall connections) of connected nodes
Rich k club, $k=\left(5,10,15,20\right)$	Fraction of edges that connect nodes of degree k or higher out of the maximum number of edges that such nodes might share

### 3.4. NPH Prediction Module: Feature Fusion

The volumes per class from the modified 3D UNet segmentation results are concatenated with the patient-specific network properties defined in Table 1. These features are then used to train a linear SVM with the L2 regularization. The output of the SVM is a binary prediction of NPH (1) or not NPH (0).

## 4. Experiments and Results

### 4.1. Segmentation Results

**Figure 5:**
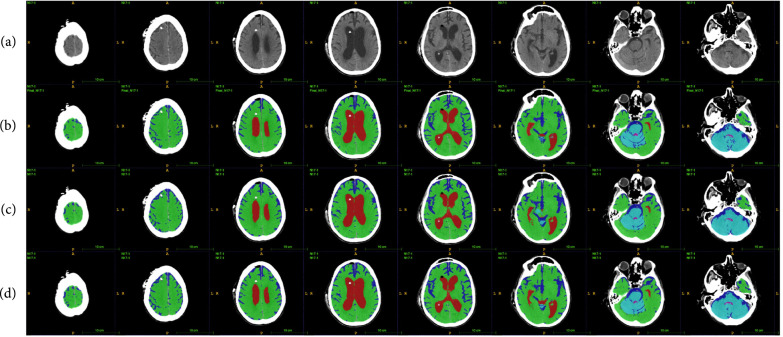
Example of segmentation results: (a) shows the original scan, (b) shows the ground truth annotations, (c) shows the results of the segmentation network without probability maps, and (d) shows the results of the segmentation network with probability maps. It can be seen that the biggest difference exists in the subarachnoid space, which takes up a small portion of the brain, but is important for clinical analysis. The slices shown are taken at every third index in the axial direction.

**Figure 6:**
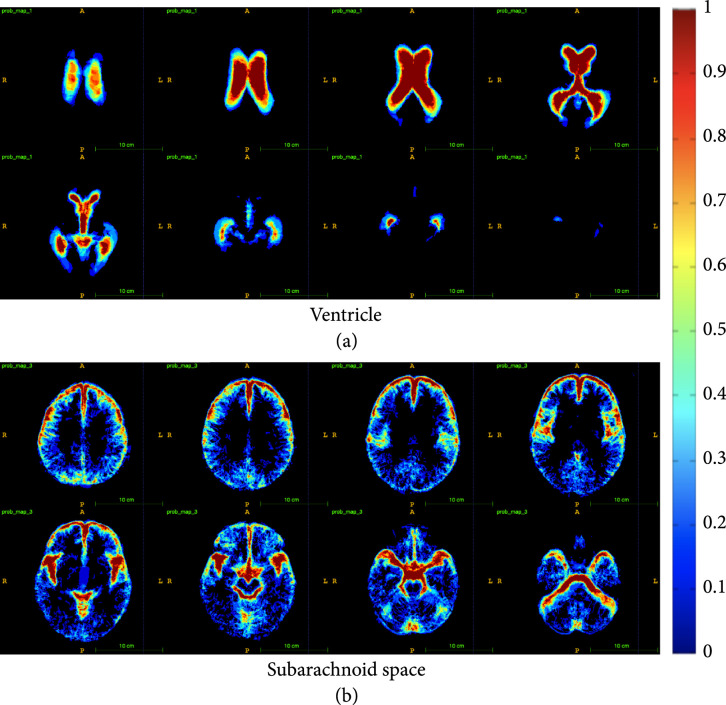
Probability maps for the ventricle (a) and subarachnoid (b) classes. Each image shows one slice in the z dimension. The progression of the images (column-wise, then row-wise) goes from the top of the brain to the bottom of the brain. Blue colors represent lower values while red colors represent higher values. Black represents a value of zero. The legend bar has been scaled to (0,1).

The modified 3D UNet with the probability map modification is trained on 30 segmented CT scans using a learning rate of 0.001, Adam optimizer, weight decay of 0.0001, dropout rate of 0.5, and hard per image cross-entropy for 300 epochs. The segmentation masks have 6 classes—the ventricle, subarachnoid space, and gray-white matter, and the same tissue classes in the cerebellum. The cerebellum is differentiated from the rest of the brain so that its volume can be observed and monitored separately for future medical studies. Fivefold cross-validation is used to verify the segmentation accuracy of the model. Example results of our segmentation algorithm are shown in Figure 5, with the generated probability maps shown in Figure 6.

Some basic machine learning methods are implemented to compare with the proposed method. They include random forest (RF) classification, 3D morphological geodesic active contours (MGAC) (Caselles et al. [23]), and 3D morphological Chan-Vese (MCV) (Chan and Vese [24]).

The implementations of these alternative methods of ventricle segmentation use thresholding to find the skull region and remove any labels outside of this region. Each implementation first computes and applies the affine transformations into MNI152 space, then computes and applies the inverse transformation after completing segmentation. For the MCV and MGAC methods, the volumes are seeded in anatomically informed locations pertaining to the gray-white matter. The regions are then grown according to their perspective algorithms.

For the scores in Table 2, the Dice score,

(1)2∣X∩Y∣∣X∣+∣Y∣=2TP2TP+FP+FN,

where X and Y are two classes (positive and negative for each class) and TP are True Positives, FP are False Positives, and FN are False Negatives, which are used for each class. The average Dice score is taken over all of the subjects. The Dice score calculates the union over intersection of a given class and is especially useful for determining the effectiveness of a segmentation algorithm when the class labels vary in size.

**Table 2:** Comparison of Dice scores for various ventricle and gray-white mass segmentation algorithms for CT scans. The scores are reported as mean±standard deviation. All methods are our implementations created for the purpose of comparison. The marked improvement in subarachnoid segmentation performance is critical for probability maps were generated from the training set for each fold.

Method	Ventricle	Gray-white matter	Subarachnoid
3D UNet+Prob. maps	85±0%	94±1%	72±5%
3D UNet	85±7%	93±1%	69±13%
RF+MCV	84±4%	87±2%	35±10%
Random forest	65±12%	87±2%	N/A
3D MGAC	25±17%	81±2%	N/A
3D MCV	13±14%	80±2%	N/A

The results of Table 2 show that our proposed method outperforms the baseline methods at segmenting the regions of interest. Our UNet without probability map enhancement also performs well in the three categories, but the use of probability maps reduces the variance in the Dice scores.

The proposed method is unique in that it allows for better separation of ventricle space and subarachnoid space. The subarachnoid space and the ventricles are both composed of cerebrospinal fluid, so they show up with similar intensities on a CT scan. Our method successfully separates these similar-looking regions with high performance.

**Figure 7:**
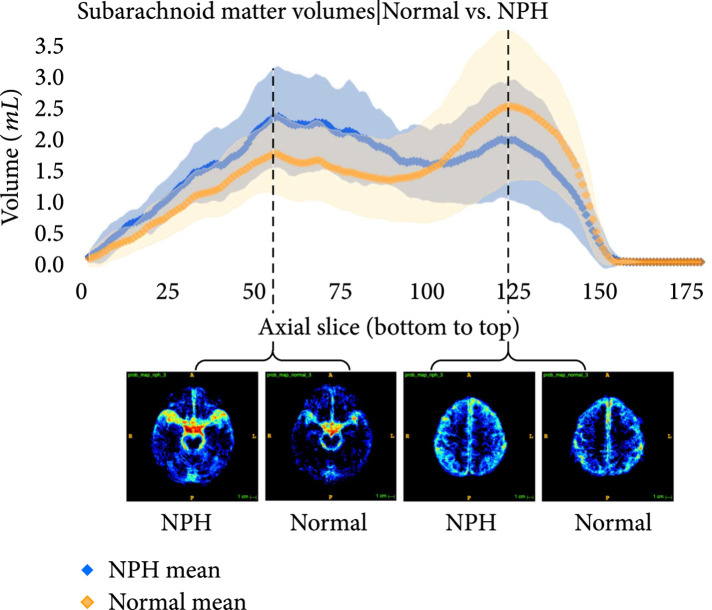
Average volumes in mL/slice of the subarachnoid in NPH (blue) and normal (orange) subjects after registration to the MNI152 space. The standard deviation is also plotted in light blue (NPH) and light orange (normal). Probability maps at inflection points are shown underneath the plot. It can be seen that the average subarachnoid space volume of subjects with NPH is greater towards the bottom of the head near the spine and smaller towards the top of the head, compared with normal subjects.

**Figure 8:**
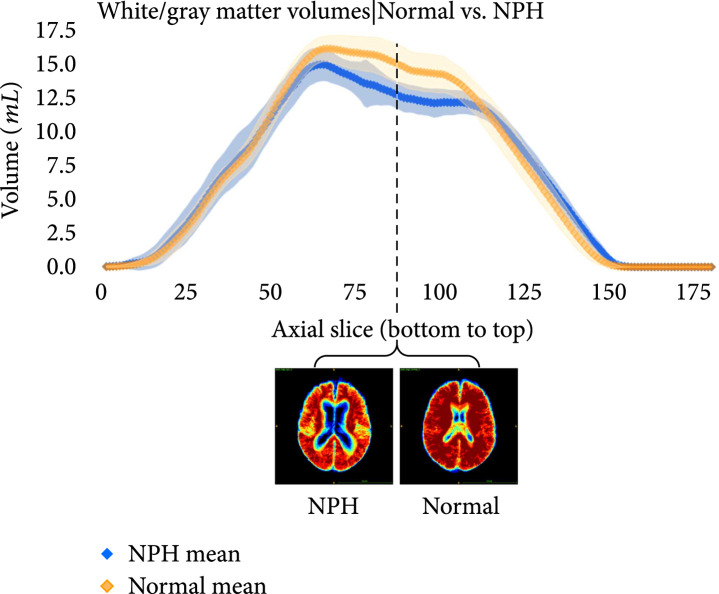
Average volumes in mL per slice of the gray-white matter in NPH (blue) and normal (orange) subjects after registration to the MNI152 space. The standard deviation is also plotted in light blue (NPH) and light orange (normal). Probability maps at the location with the largest difference between the means is shown underneath the plot.

**Figure 9:**
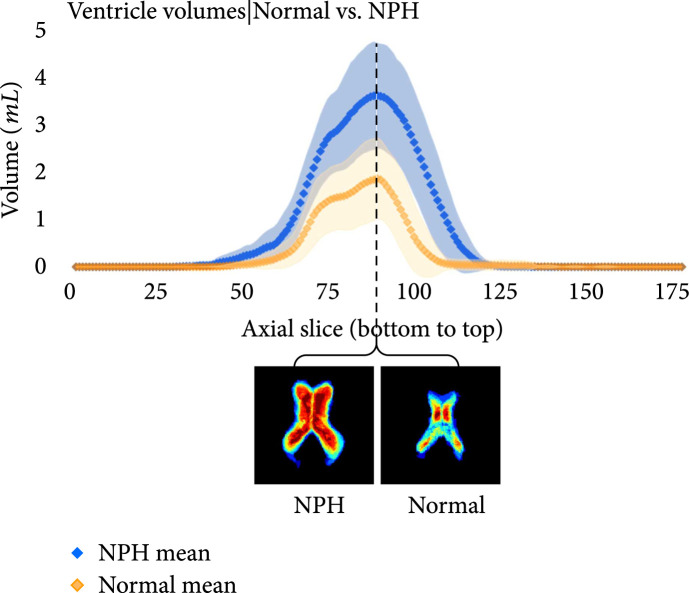
Average volumes in mL per slice of the ventricles in NPH (blue) and normal (orange) subjects after registration to the MNI152 space. The standard deviation is also plotted in light blue (NPH) and light orange (normal). Probability maps at the location with the largest difference between the means is shown underneath the plot. As expected, the volumes are greater for subjects with NPH than those without NPH.

**Figure 10:**
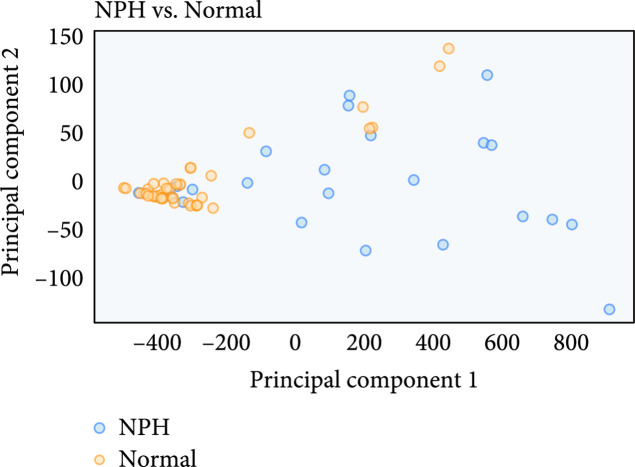
Average location of network metric after PCA reduction from 26 connectivity features to 2 principal components. For the most part, a separation can be seen between normal and NPH subjects, but there is some mixing of the classes as well. The trend towards separation shows why the network properties are useful features for NPH prediction.

The following plots further analyze the results of the segmentations for NPH and normal subjects. Figures 7–9 show the mean and standard deviation of volumes of each region by slice in the axial direction. Figure 10 shows the distribution of subjects by diagnosis based on their connectivity network properties.

### 4.2. NPH Prediction and Comparison with Evan’s Ratio

For comparison, each scan is labeled with Evan’s ratio as measured under direct supervision of a neurological surgeon. NPH predictions on the labeled subset using only Evan’s ratio are first computed by the current guidelines, with subjects having Evan’s ratio greater than or equal to 0.3 classified as NPH, and the remaining subjects classified as non-NPH.

Precision and recall are calculated for 3 methods of NPH prediction—thresholding of the manually annotated Evans’ index, using fully connected layers with the modified 3D UNet features, and using fully connected layers with the modified 3D UNet features along with the network properties.

Precision is defined as the number of true predicted positives over the number of all predicted positives. Recall is defined as the number of true predicted positives over the total number of actual positives. Essentially, precision is a proxy for how many selected elements are relevant, while recall is a proxy for how many relevant elements are selected.

As seen in Table 3, using the volumetric information and network properties for prediction of NPH outperformed Evans’ index thresholding in both precision and recall. We did not implement automated Evans’ index calculations from Takahashi et al. [5] for comparison purposes, because the paper claims equivalence to manual Evans’ index calculations as the best case scenario. One-sided t-tests yielded p<0.001 for precision and recall between model 3 and model 2 and p<0.1 for precision and recall between model 3 and model 1. Performance did not vary when we used the 3D UNet model without probability maps. This is expected, for the purpose of the probability maps is to achieve higher accuracy in the finer details of the segmentation for clinical analysis purposes.

**Table 3:** NPH prediction scores using various methods and features. All rows except the first row (Evan’s index) used a linear support vector machine for training and testing. The predictive models are trained and tested for 100 iterations using 5-fold cross-validation with randomized selection at each fold using scikit-learn, as explained in Pedregosa et al. [25].

	Precision (train/test)	Recall (train/test)
Evan’s index, thresholding	86	70
Volumetric features (model 1)	86±3/86±13	80±7/76±17
Network properties (model 2)	80±5/78±17	85±7/75±21
Volumetric features+network properties (model 3)	96±3/93±12	89±4/89±13

**Figure 11:**
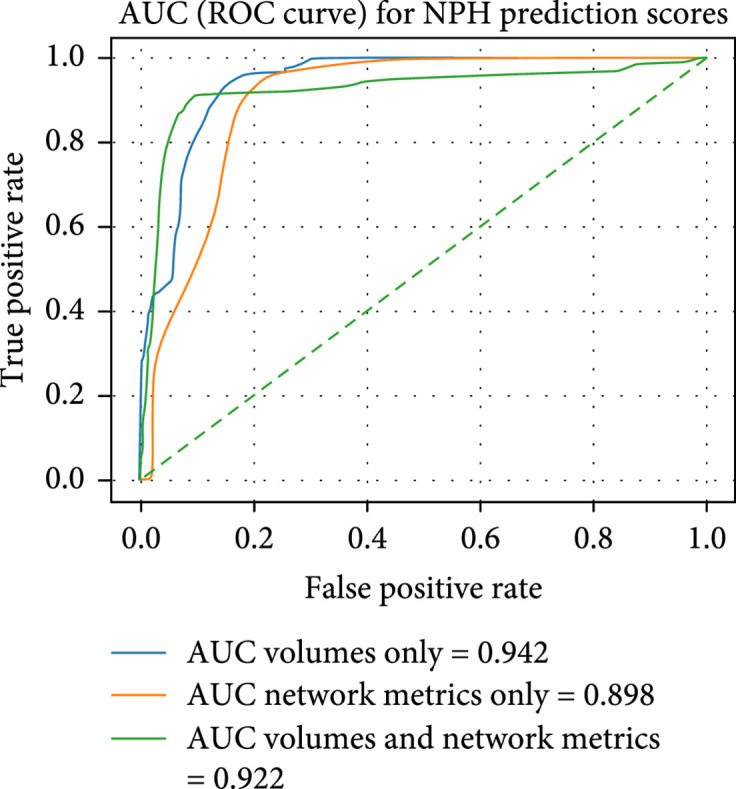
Test AUC for the linear SVM trained on different features: ventricle, subarachnoid, gray-white matter, and overall volumes (4 features); network properties (26 features); and volume and network properties (30 features). While the AUC for volumes and network metrics is lower than that for volumes only, the inflection point of the model associated with volumes and network metrics has consistently higher performance over 100 iterations using 5-fold validation. Each iteration uses a new generator for the 5-folds.

**Figure 12:**
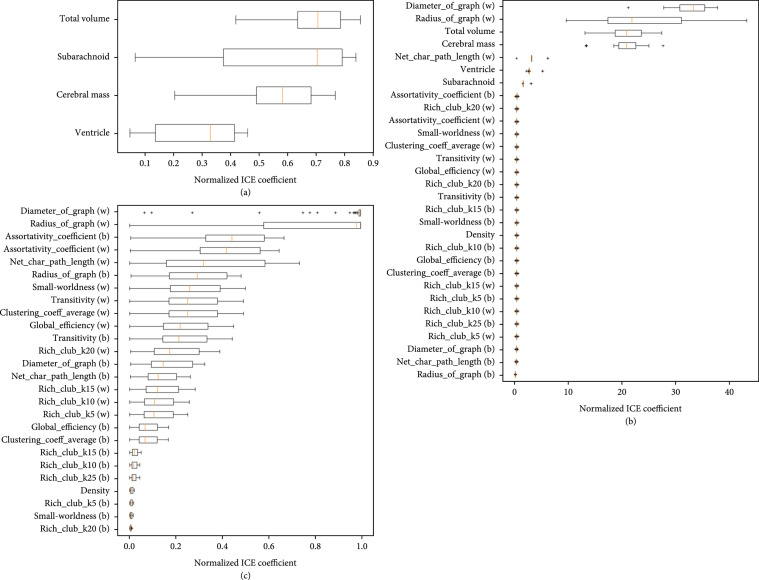
Feature significance of each SVM model using the ML-insights python package. (a) shows the importance of each feature when only volumetric features are used. (b) shows the importance of each feature when only network properties are used. (c) shows the importance of each feature when volumetric and network properties are used. It is interesting to see that in (c), the most important features are a mixture of volumetric features and network properties. For (a) and (c), linear scale was used, while in (b), log scale was used.

We plot the AUC and feature importance of the SVM in Figures 11 and 12.

Feature importance was calculated by using 5-fold cross-validation to generate class membership probabilities using logistic regression on SVM’s scores (Platt and Karampatziakis [26]). After obtaining the class membership probabilities, individual conditional expectation plots (Goldstein et al. [27]) are generated using the ML-insights software. From Figure 12, it can be seen that the most important features when predicting NPH with volume information only is the total volume and the subarachnoid space. For plots (b) and (c), it can be seen that the diameter and radius of the graph are important network metrics for predicting NPH. When all of the features are used for NPH prediction, a mixture of volumetric and network features are deemed important. All of the volumetric features were in the top 30% of the most important features for NPH prediction.

## 5. Discussion and Future Work

### 5.1. Summary

The paper presents a fully automated, volumetric method of lateral ventricles, subarachnoid space, and gray-white mass segmentation in CT scans. Additionally, this paper proposes a fully automated method to predict NPH diagnosis, which, in conjunction with the clinical symptomatology, can facilitate the diagnosis of NPH and rule out subjects who do not meet the radiographic criteria of an NPH diagnosis. This technological system outperforms the thresholding method using Evan’s ratio and can be used as a screening tool to identify or stratify possible NPH cases in a clinical setting. The segmentation model for this paper can be used to segment and study the volumes of CT brain scans in cases other than NPH.

Furthermore, this paper contributes a novel method of using network properties to aid in NPH diagnosis prediction and explores potential brain regions most affected by NPH by studying the connectivity changes that may occur during ventricle dilation.

### 5.2. Study Limitations and Future Work

The work presented in this paper is intended as a proof of concept, with representative samples of CT scans from subjects in each category. The network is sensitive to differences in CT appearance due to age, so a fusion of networks may be used in the future to accommodate patients of all ages, not only those in the NPH risk range. Additionally, it would be beneficial to provide more examples of CT scans with high resolution in the sagittal and coronal planes, to make the model robust to scanning direction. One intrinsic limitation of this paper is the idiopathic nature of NPH, meaning it is diagnosed when other conditions have been ruled out. This could lead to a biased dataset. We would like to include more confounding factors in future studies, such as the presence of Alzheimer’s, Parkinson’s, or non-NPH hydrocephalus.

Currently, the runtime for diffusion tractography and network analysis takes approximately 1.5 minutes per subject on a standard computer with 64 GB of RAM. It is possible to explore alternatives to the tractography process using methods such as probabilistic tractography, as described in (Sarwar et al. [28]) or tractography using deep learning methods, as presented in (Poulin et al. [29]; Tian et al. [30]; Benou and Raviv [31]).

To conclude, autosegmentations of CT scans are helpful because CT scans are more common, readily available, and accessible compared to MRI scans. Since autosegmentations of CT scans have not been done before in such detail, this machine-learning-based algorithm can be applied to study many other neurological conditions. In particular, changes over time with serial CT scans can be examined both retrospectively and prospectively. For example, CT scans of patients with Alzheimer’s disease, TBI, or NPH can be analyzed to compare the ratios of ventricles to subarachnoid to the cerebrum. Changes in these ratios can hold valuable information about the progression/resolution of these disease entities. Furthermore, with respect to NPH, this ability to do this type of detailed volumetric analysis space offers tremendous potential for determining whether or not there are changes reflective of proper shunt functioning.

## Data Availability

The computed tomography data used to support the findings of this study are available from the corresponding author upon request.
